# Intestinal microbiome in children with severe and complicated acute viral gastroenteritis

**DOI:** 10.1038/srep46130

**Published:** 2017-04-11

**Authors:** Shih-Yen Chen, Chi-Neu Tsai, Yun-Shien Lee, Chun-Yuan Lin, Kuan-Yeh Huang, Hsun-Ching Chao, Ming-Wei Lai, Cheng-Hsun Chiu

**Affiliations:** 1 Division of Pediatric Gastroenterology, Chang Gung Children’s Hospital, Chang Gung University College of Medicine, Taoyuan, Taiwan; 2 Graduate Institute of Clinical Medical Sciences, Chang Gung University College of Medicine, Taoyuan, Taiwan; 3 Department of Biotechnology, Min-Chuan University, Taoyuan, Taiwan; 4 Department of Computer Science and Information Engineering, Chang Gung University College of Engineering, Taoyuan, Taiwan; 5 Molecular Infectious Disease Research Center, Chang Gung Memorial Hospital, Chang Gung University College of Medicine, Taoyuan, Taiwan

## Abstract

The aim of the present study was to evaluate the microbiota of children with severe or complicated acute viral gastroenteritis (AGE). To that end, next-generation sequencing (NGS) technology was used to sequence the 16S ribosomal RNA (*16S rRNA*) gene in 20 hospitalized pediatric patients with severe or complicated AGE and a further 20 otherwise healthy children; the fecal microbiome was then assessed. Comparative metagenomics data were analyzed by a Wilcoxon rank–sum test and hierarchical clustering analysis of bacterial reads. The statistical analyses showed a significantly decreased Shannon diversity index (entropy score) of the intestinal microbiota in patients with severe AGE compared with normal controls (*P* = 0.017) and patients with mild-to-moderate AGE (*P* = 0.011). The intestinal microbiota score of the 5 patients with rotavirus AGE was significantly lower than that of those with norovirus infection (*P* = 0.048). Greater richness in *Campylobacteraceae (P* = 0.0003), *Neisseriaceae (P* = 0.0115), *Methylobacteriaceae (P* = 0.0004), *Sphingomonadaceae (P* = 0.0221), and *Enterobacteriaceae (P* = 0.0451) was found in patients with complicated AGE compared with normal controls. The data suggest a significant reduction in intestinal microbial diversity in patients with severe AGE, particularly those with rotavirus infection.

Intestinal microorganisms are related to human health; therefore, recent research has focused on intestinal microflora and its association with human disease. Moreover, study of the intestinal microbiota of infants is important, as it undergoes rapid changes. The interactions of the intestinal microflora with the host have been investigated using next-generation sequencing (NGS) technologies^1^,^2^,^3^. NGS has had a major impact on genome-wide screening of diseases, microbial metagenomics, and validation of undetermined human pathogens^4^,^5^,^6^,^7^.

An earlier study investigated the effect of medical procedures on the intestinal microbiota and its relationship to health and disease in neonates^8^. The genetic and environmental interactions of the intestinal microbiota are reportedly correlated with the progression of inflammatory bowel disease^9^. Acute viral gastroenteritis (AGE) can be associated with atypical manifestations, such as convulsions and intestinal hemorrhage^10^,^11^,^12^. Recent studies found that the adaptive responses against commensals consist integral component of mucosal immunity in gastrointestinal infection, and following norovirus infection, patients who have a disrupted microbiota may be at elevated risk for long-term health complications^13^,^14^.

However, the role of the intestinal microbiota in AGE, and the underlying mechanisms, have not been determined. In the present study, NGS was used to evaluate the intestinal microbiota of children with severe or complicated AGE.

## Results

### Patient enrollment and 16S ribosomal RNA (*16S rRNA*) sequencing

A total of 40 stool samples were collected, comprising 20 fecal samples from pediatric patients with AGE (mean age, 16.9 months; range, 0–91 months) and 20 stool samples from disease-free infants or children (negative controls; mean age, 17.4 months; range, 0–54 months). Of the 20 patients, 15 patients had a norovirus infection and 5 had a rotavirus infection, including 15 with severe AGE (Vesikari score >10)^15^ and 5 with mild-to-moderate AGE (Vesikari score ≦ 10). The 15 complication presentations comprised 10 AGE with convulsion (7 norovirus, 3 rotavirus infections), 2 necrotizing enterocolitis (norovirus infections), 2 gastroenteritis with renal failure or severe electrolyte imbalance (norovirus infections), and 1 gastroenteritis with chronic diarrhea of prolonged duration (>3 weeks) and malnutrition (norovirus infection). Antibiotics were administered in 2 infants with necrotizing enterocolitis before surgery and a further 1 infant with renal failure. All fecal samples were collected at least 1 week after discontinuation of the antibiotics. Enteric bacteria in the 40 fecal samples were classified into 9 phyla, 62 families, and 159 genera based on *16S rRNA* sequences and metagenomic analysis of all selected reads.

### Diversity of the intestinal microbiota in children with severe gastroenteritis

**Figure 1:**
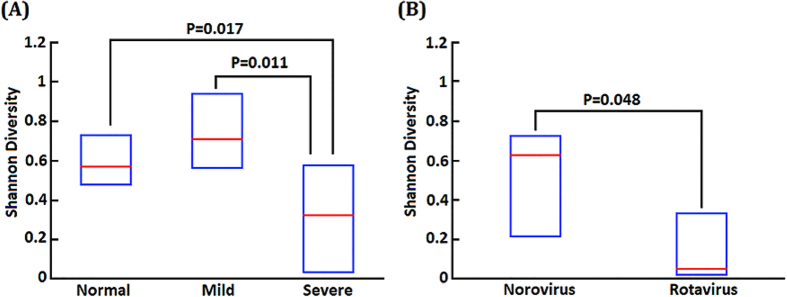
Diversity of microbiota among healthy controls and AGE patients with different severity. Statistical analysis showed significantly decreased Shannon diversity (entropy score) of intestinal microbiota in severe viral AGE, when compared to normal controls (*P* = 0.017) and to patients with mild to moderate severity (*P* = 0.011) (**A**). The entropy score of the rotavirus infection was 0.05 (0.02–0.35), that was significantly lower than norovirus infection group (*P* = 0.048) (**B**).

The Shannon diversity indices of the fecal samples were calculated and reported as entropy scores^16^. These are indicative of the intestinal microbiota composition and disruption of the microbiota, as scores are lower in the presence of overgrowth of a single bacterial species. The entropy score of the intestinal microbiota of the normal controls, and patients with mild-to-moderate and severe AGE were 0.56 (0.47–0.73), 0.7 (0.56–0.93), and 0.3 (0.05–0.6), respectively (Fig. 1A). The Shannon diversity index (entropy score) of the intestinal microbiota was significantly lower in patients with severe AGE than in normal controls (*P* = 0.017) and patients with mild-to-moderate AGE (*P* = 0.011).

The average severity score of the 5 patients with rotavirus infection was 13.8 (range, 11–15), which is significantly higher than that of the 15 patients with norovirus infection (mean, 10.3; range, 8–11) (*P* = 0.03). The entropy score in patients with norovirus infection was 0.65 (0.2–0.75), and was not significantly different compared with that of the normal controls. The entropy score of patients with rotavirus infection was 0.05 (0.02–0.35), which was significantly lower than that of patients with norovirus infection (*P* = 0.048) (Fig. 1B).

**Figure 2:**
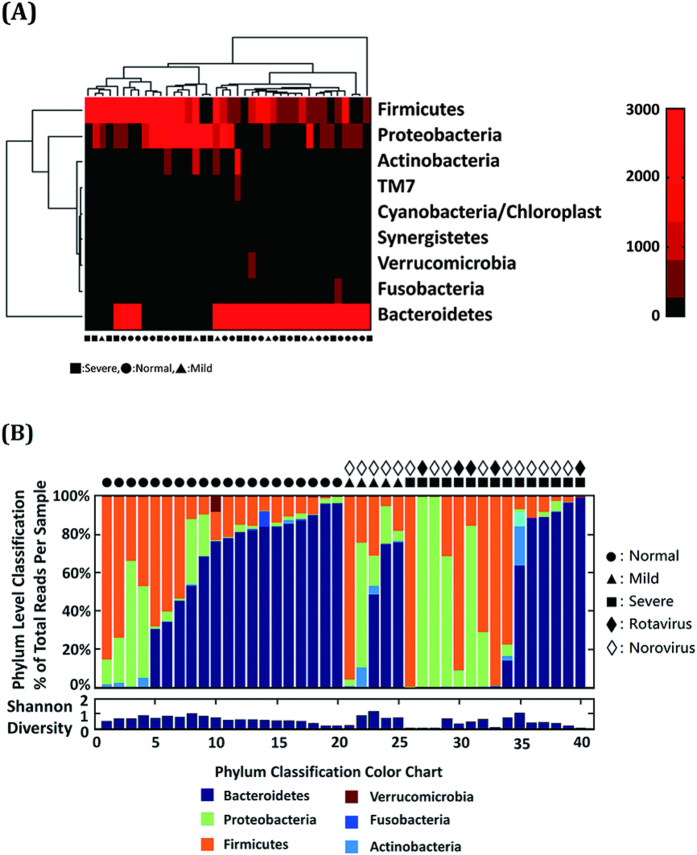
Intestinal microbiota in family level in healthy controls and AGE patients with various severity and caused by rotavirus or norovirus. The intestinal microbiota distrubtion in phylum level of normal control, mild, and servere disease groups of acute viral gastroenteritis (**A**). The microbiota distribution in patients with rotavirus or norovirus infections and their disease severity classifications (**B**).

The intestinal microbiota composition at the phylum level of normal controls, and patients with mild-to-moderate and severe AGE is shown in Fig. 2A. The microbiota composition in patients with rotavirus or norovirus infection and the associated disease severity classification are shown in Fig. 2B. There was no significant difference in microbiota composition between the normal controls and patients with AGE at the phylum level. The feces of normal controls exhibited significant abundance of *Rikenellaceae (P* = 0.043) and *Porphyromonadaceae (P* = 0.02) at the family level, and *Alistipes (P* = 0.038) and *Parabacteroides (P* = 0.019) at the genus level compared with any severity of AGE patients.

### Clinical correlation

The correlations of the intestinal microbiota composition with clinical manifestations were evaluated (Table 1). At the family level, patients with abdominal pain exhibited greater abundance of *Prevotellaceae (P* = 0.013), *Staphylococcaceae (P* = 0.013), and *Coriobacteriaceae (P* = 0.015). *Veillonellaceae (P* = 0.048) abundance was lower in patients hospitalized for longer than 7 days. The abundance of *Micrococcaceae (P* = 0.011) and *Campylobacteraceae (P* = 0.05) was lower in patients with extraintestinal manifestations of viral infection. At the genus level, patients with abdominal pain had greater richness in *Prevotella (P* = 0.013), *TM7 (P* = 0.013), *Staphylococcus (P* = 0.013), and *Atopobium (P* = 0.017). Patients with convulsion showed absence of *Haemophilus (P* = 0.045) and with substantially decreased genus of *Faecalibacterium (P* = 0.059).

We also analyzed the intestinal microbiota composition of 3 patients with complicated AGE, comprising 1 patient with necrotizing enterocolitis after norovirus infection, 1 with rotavirus infection with convulsions, and 1 with norovirus infection and renal failure (Supplementary Fig. 1). In the norovirus infection patient with necrotizing enterocolitis, the entropy score of the intestinal microbiota was 0.008833, and *Streptococcacea*e (63%), *Enterobacteriaceae* (23%), and *Pasteurellaceae* (6%) comprised >90% of the microbiota. The entropy score of patient with rotavirus infection and convulsions was 0.02779, and *Bacteroidaceae* comprised almost 100% of the intestinal microbiota in this patient. The entropy score of norovirus infection patient with renal failure was 0.005949, and *Enterococcaceae, Clostridiaceae,* and *Streptococcaceae* comprised >95% of the microbiota.

### Microbiota of viral AGE patients with and without complications

There were 15 children that experienced complications after viral AGE and 5 without complications. The intestinal microbiota of the children with viral AGE differed from that of healthy controls at the family level (Table 2). The overall differences in healthy children, and those with non-complicated and complicated AGE were in the richness of *Porphyromonadaceae* in healthy children (*P* = 0.035) and *Streptococcaceae* in uncomplicated AGE (*P* = 0.035), shown by the composition of fecal bacteria. Patients with complicated AGE had greater abundance of Campylobacteraceae (*P* = 0.0003), *Neisseriaceae (P* = 0.0115)*, Methylobacteriaceae (P* = 0.0004)*, Sphingomonadaceae (P* = 0.0221), and *Enterobacteriaceae (P* = 0.0451) than healthy children. Uncomplicated AGE was associated with a decrease in the abundance of *Desulfovibrionaceae (P* = 0.0198), *Ruminococcaceae (P* = 0.0125)*, Veillonellaceae (P* = 0.0199) and increase in *Carnobacteriaceae (P* = 0.0124). Finally, patients with complicated AGE had greater *Pasteurellaceae* richness (*P* = 0.0071) than patients with AGE without complications.

## Discussion

The intestinal microbiota diversity was less in patients with severe viral AGE compared with healthy controls and those with mild-to-moderate AGE, suggesting the counterpoise of microbiota being changed or disrupted into some overgrowing bacterial species. Recent studies suggested that disruption of the gut microbiota is associated with antibiotic use following viral infections^13^,^14^,^17^. Rotavirus infection resulted in decreased microbiota diversity compared with norovirus infection, which has not to our knowledge been reported previously although in our study, the mean severity of rotavirus AGE is higher. Recent studies showed that norovirus infection of B cells *in vivo* was facilitated by bacteria expressing an appropriate histo-blood group antigen^18^,^19^. This could explain the difference in microbiota composition between patients with rotavirus and norovirus infections.

Richness or reduction in specific families or genera was related to the presence of abdominal pain, longer hospitalization, multi-system involvement, and convulsion, but not diarrhea, vomiting, or fever also gender and age. An association between intestinal symptoms and the intestinal microbiota, as well as a significant inverse correlation between *Bifidobacteria* and abdominal pain, has been reported in adults^20^; no such association was found in children in this study. Nevertheless, our results showed that several bacterial taxa—such as *Prevotellaceae, Staphylococcaceae,* and *Coriobacteriaceae*—were more abundant in children with abdominal pain. The relative instability of the intestinal microbial community in children might explain this discrepancy.

Another study reported that patients with irritable bowel syndrome had significantly higher counts of *Veillonella* than controls, which appeared to be associated with high acetic acid or propionic acid levels in the bowel^21^. Probiotic supplementation could stabilize the microbiota and alleviate symptoms of irritable bowel syndrome^22^,^23^. However, this was not the case in our study: the major features of patients with abdominal pain were overgrowth of particular bacterial species, and no deficiency in specific microbial taxa was evident. Therefore, supplementation should not be recommended for AGE patients with significant abdominal pain.

The microbiota of stool specimens from patients with complicated AGE exhibited greater richness either at the phylum, family, or species level, suggesting that several important gut species remain to be characterized. The characterization offers a new approach to identifying individuals at high risk of developing specific complications following viral AGE. During early life, the intestinal microbiota changes with age^24^. In some cases of complicated viral AGE, such as necrotizing enterocolitis following norovirus infection, rotavirus infection and convulsions, and norovirus infection complicated by renal failure, extremely lower microbiota diversity was found. This suggests that these complications could be associated with disruption of the microbiota.

When compared with the intestinal microbiota in healthy early life^8^,^24^, the dominant taxa in complicated AGE were *Streptococcaceae, Enterobacteriaceae*, and *Pasteurellaceae* rather than *Bifidobacteriaceae, Clostridiaceae,* and *Lactobacillaceae* in a representative necrotizing enterocolitis patient; *Bacteroidaceae, Lachnospiraceae*, and *Porphyromonadaceae* rather than *Clostridiaceae, Lachnospiraceae*, and *Bifidobacteriaceae* in a representative patient with convulsions; and *Enterococcaceae, Clostridiaceae*, and *Streptococcaceae* rather than *Clostridiaceae, Lachnospiraceae*, and *Ruminococcaceae* in a representative acute renal failure patient. Our data showed that, in addition to antibiotic use (which may influence the microbiota composition), viral infection might also affect the diversity and predominant taxa of the intestinal microbiota, particularly in patients with severe disease or complications.

The use of probiotics such as *Lactobacillus rhamnosus GG* and *Saccharomyces boulardii,* or the combination of *Streptococcaceae faecalis, Clostridium butyricum,* and *Bacillus mesentericus* to treat or prevent AGE has been evaluated^25^,^26^. In the present study, *Streptococcaceae* richness was significantly greater in patients with uncomplicated compared with complicated AGE, and *Parabacteroides* and *Porphyromonadaceae* abundance was greater in healthy controls than in patients with severe AGE and in the complicated ones individually and this would trigger more follow-up investigations for future clinical application.

This study had several limitations. First, a limited number of cases were evaluated. Second, use of antibiotics by some of the patients prior to confirmation of a viral etiology could have affected the intestinal microbiota. Third, acute clinical symptoms and intestinal microbiota composition were investigated at a single time point; no longitudinal follow-up was performed.

In conclusion, we evaluated the intestinal microbiota composition of children with viral AGE and its correlations with various severities also with or without complications. The results will facilitate further studies of the interaction between the intestinal microbiota and viral pathogens, and use of probiotics to treat AGE caused by rotavirus or norovirus.

## Methods

### Ethics

This study was approved by the Institutional Review Board of Chang Gung Memorial Hospital (CGMH100-4283A3), and the participants or their guardians provided informed consent for specimen and clinical data collection. All applied methods were performed in accordance with the approved guidelines.

### Patient enrollment and sample collection

Patients less than 18 years old hospitalized at Chang Gung Children’s Hospital from August 2012 to July 2013 whose major clinical manifestation was AGE and who fulfilled the inclusion criteria were offered enrollment. All medical records of enrolled patients were reviewed for the clinical and demographic data. We evaluated the severity of gastroenteritis using the Vesikari score system^15^. A total score of >10 indicates severe gastroenteritis (maximum total score 20, total score <7, mild; 7 ≤ 10, moderate; and >10, severe). Children with gastroenteritis symptoms and any one of the following clinical complications or conditions were included in the complicated gastroenteritis group: extraintestinal or unusual presentations of viral gastroenteritis such as electrolyte imbalance, renal failure, shock, convulsive disorder, prominent hyperthermia (>39 °C), hypotension or hypovolemic shock (systolic blood pressure <70 mmHg), hypoglycemia (serum sugar <70 mg/dL), chronic diarrhea (>3 weeks) resulting in malnutrition, and necrotizing enterocolitis. Involvement of multiple systems indicated involvement of systems other than the gastrointestinal tract. Twenty otherwise healthy children were enrolled as negative controls. Their fecal specimens were collected within 3 d of hospitalization or complication events and were stored at −70 °C before extraction.

### Extraction of stool DNA

Stool total DNA was extracted using a QIAamp^®^ DNA Stool Mini Kit (Qiagen, Hilden, Germany) according to the manufacturer’s recommendations including the procedure of heating suspension for 5 min at high temperature of 95 °C and this can help to lyse Gram positive bacteria and was stored at −80 °C^27^. Universal primers for the *16S* variable regions V1–3 and V3–5 were used for polymerase chain reaction (PCR) amplification. The V3–534 and V5–926 reverse primers included a unique sequence tag to barcode the samples (http://www.hmpdacc.org/doc/16S_Sequencing_SOP_4.2.2.pdf).

### NGS

The purified amplicon mixtures were sequenced using the 454 GS Junior System according to the protocols recommended by the manufacturer (454 Life Sciences, Branford, CT, USA). The regions of *16S rRNA* genes were amplified using FastStart™ HiFi Polymerase (Roche, Manheim, Germany). The PCR amplicons were purified using Agencourt^®^ AMPure^®^ XP Reagent (Beckman Coulter, Brea, CA, USA) and quantified using an Agilent Bioanalyzer (Agilent Technologies, Santa Clara, CA, USA).

### Statistical and comparative genomics analyses

To analyze the data, ~1 × 10^5^ reads not aligned to the human genome were randomly selected from all reads with an average length of ~100 base pairs, and similar sequences were searched for in the NCBI nt (National Center for Biotechnology Information nucleotide) database using BLASTn (Basic Local Alignment Search Tool nucleotide database) with a threshold e-value of 5 × 10^−5^. The full-length reference genome was used for complete genome analysis. Sequences were aligned using the CLUSTAL W software included in MEGA v. 5.0 (http://mega.software.informer.com/5.0/) and the bootstrap was plotted for a window of 300 bp, moving in increments of 10 bp along the alignment, using Simplot software (http://sray.med.som.jhmi.edu/SCRoftware/simplot/). All reference sequences used in this study were obtained from GenBank (http://www.ncbi.nlm.nih.gov/genbank/). The fecal microbiota composition was characterized compared to microbiota across body sites in the Human Microbiome Project (HMP) database (https://commonfund.nih.gov/hmp/index).

Continuous clinical data were analyzed using Student’s *t*-tests and expressed as means ± standard deviation (SD). Binary data were analyzed using the χ^2^ test. A value of *P* < 0.05 was considered to indicate statistical significance. All tests were performed using SAS software v. 8 for Windows (SAS Institute Inc., Cary, NC, USA).

## Additional Information

**How to cite this article**: Chen, S.-Y. *et al*. Intestinal microbiome in children with severe and complicated acute viral gastroenteritis. *Sci. Rep.* **7**, 46130; doi: 10.1038/srep46130 (2017).

**Publisher's note:** Springer Nature remains neutral with regard to jurisdictional claims in published maps and institutional affiliations.

## Supplementary Material

Supplementary Information

## Figures and Tables

**Table 1:** Different Intestinal Microbiota at Family and Genus Level Assignments by Clinical Symptoms.

Clinical symptoms and intestinal microbiota	No	Yes	P value
**Family level**			
**Abdominal pain (n.)**	**6**	**14**	
* Prevotellaceae*	0.006% ± 0.01%	30% ± 52%	0.013
* Staphylococcaceae*	0.004% ± 0.015%	0.9% ± 1.6%	0.013
* Coriobacteriaceae*	0.04% ± 0.11%	1% ± 2%	0.015
**Longer hospitalization (** 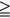 **7 days) (n.)**	**11**	**9**	
* Veillonellaceae*	15% ± 23%	0.5% ± 0.8%	0.048
**Involvement of multiple systems(n.)**	**8**	**12**	
* Micrococcaceae*	0.3% ± 0.4%	0.006% ± 0.018%	0.011
* Campylobacteraceae*	3% ± 5%	0.004% ± 0.013%	0.05
**Genus level**			
**Abdominal pain (n.)**	**6**	**14**	
* Prevotella*	0.006% ± 0.011%	30% ± 52%	0.013
* TM7*	0.0008% ± 0.0032%	3% ± 5%	0.013
* Staphylococcus*	0.005% ± 0.020%	0.8% ± 1.4%	0.013
* Atopobium*	0.03% ± 0.12%	0.98% ± 1.69%	0.017
**Convulsion (n.)**			
* Haemophilus*	0.4% ± 0.7%	0% ± 0%	0.045

n. number.

**Table 2:** Intestinal Microbiota (average reads) in Healthy Children and Those with Acute Gastroenteritis with or without Complications.

Fecal Microbiota	AGE with complication (n = 15)	AGE without complication (n = 5)	Healthy children (n = 20)	*P*-value
*Porphyromonadaceae*	0	43.5625	326.55	0.0346^a^
*Campylobacteraceae*	178.4	0.1875	0	0.0003^b^
*Desulfovibrionaceae*	0	0	5.1	0.0198^c^
*Neisseriaceae*	4	0.5	0.45	0.0115^b^
*Phyllobacteriaceae*	0	0.1875	0	0.0346^a^
*Methylobacteriaceae*	0.8	25.5625	0	0.0004^b^
*Sphingomonadaceae*	0.8	31.5625	0.2	0.0221^b^
*Enterobacteriaceae*	2676.4	1523.5	804.7	0.0451^b^
*Pasteurellaceae*	70.2	25.6875	2.4	0.0071^d^
*Ruminococcaceae*	22	53.4375	220.8	0.0125^c^
*Streptococcaceae*	213.8	801.3125	166.75	0.0351^a^
*Carnobacteriaceae*	1.2	30.875	0.2	0.0124^c^
*Veillonellaceae*	1385.6	308.5	859	0.0199^c^

^a^Statistically significant among 3 categories.

^b^Statistically significant between AGE with complication and healthy children.

^c^Statistically significant between AGE without complication and healthy children.

^d^Statistically significant between AGE with and without complication.
