# Corrigendum: Intestinal microbiome in children with severe and complicated acute viral gastroenteritis

**DOI:** 10.1038/srep46820

**Published:** 2017-05-26

**Authors:** Shih-Yen Chen, Chi-Neu Tsai, Yun-Shien Lee, Chun-Yuan Lin, Kuan-Yeh Huang, Hsun-Ching Chao, Ming-Wei Lai, Cheng-Hsun Chiu

Scientific Reports 7: Article number: 46130; 10.1038/srep46130 published online: 04 11 2017; updated: 05 26 2017.

In this Article, an affiliation for Chi-Neu Tsai was omitted. The correct affiliations for Chi-Neu Tsai are listed below:

Division of Pediatric Gastroenterology, Chang Gung Children’s Hospital, Chang Gung University College of Medicine, Taoyuan, Taiwan.

Graduate Institute of Clinical Medical Sciences, Chang Gung University College of Medicine, Taoyuan, Taiwan.

