# Supplementary material for: Intestinal microbiome in children with severe and complicated acute viral gastroenteritis
Source: Sci Rep. 2017 Apr 11;7:46130. doi: 10.1038/srep46130 (PMC5387401; doi:10.1038/srep46130)
Supplement: Supplementary Information [file srep46130-s1.pdf]

Supplemental informations for

Intestinal microbiome in children with severe and complicated acute viral Gastroenteritis

Shih-Yen Chen[1], Chi-Neu Tsai[2], Yun-Shien Lee[3], Chun-Yuan Lin[4], Kuan-Yeh Huang[5], Hsun-Ching Chao[1], Ming-Wei Lai[1] & Cheng-Hsun Chiu[2,5,*]

[1]Division of Pediatric Gastroenterology, Chang Gung Children's Hospital, Chang Gung University College of Medicine, Taoyuan, Taiwan.

[2]Graduate Institute of Clinical Medical Sciences, Chang Gung University College of Medicine, Taoyuan, Taiwan

[3]Department of Biotechnology, Min-Chuan University, Taoyuan, Taiwan

[4]Department of Computer Science and Information Engineering, Chang Gung University College of Engineering, Taoyuan, Taiwan

[5]Molecular Infectious Disease Research Center, Chang Gung Memorial Hospital, Chang Gung University College of Medicine, Taoyuan, Taiwan.

sFig. 1A

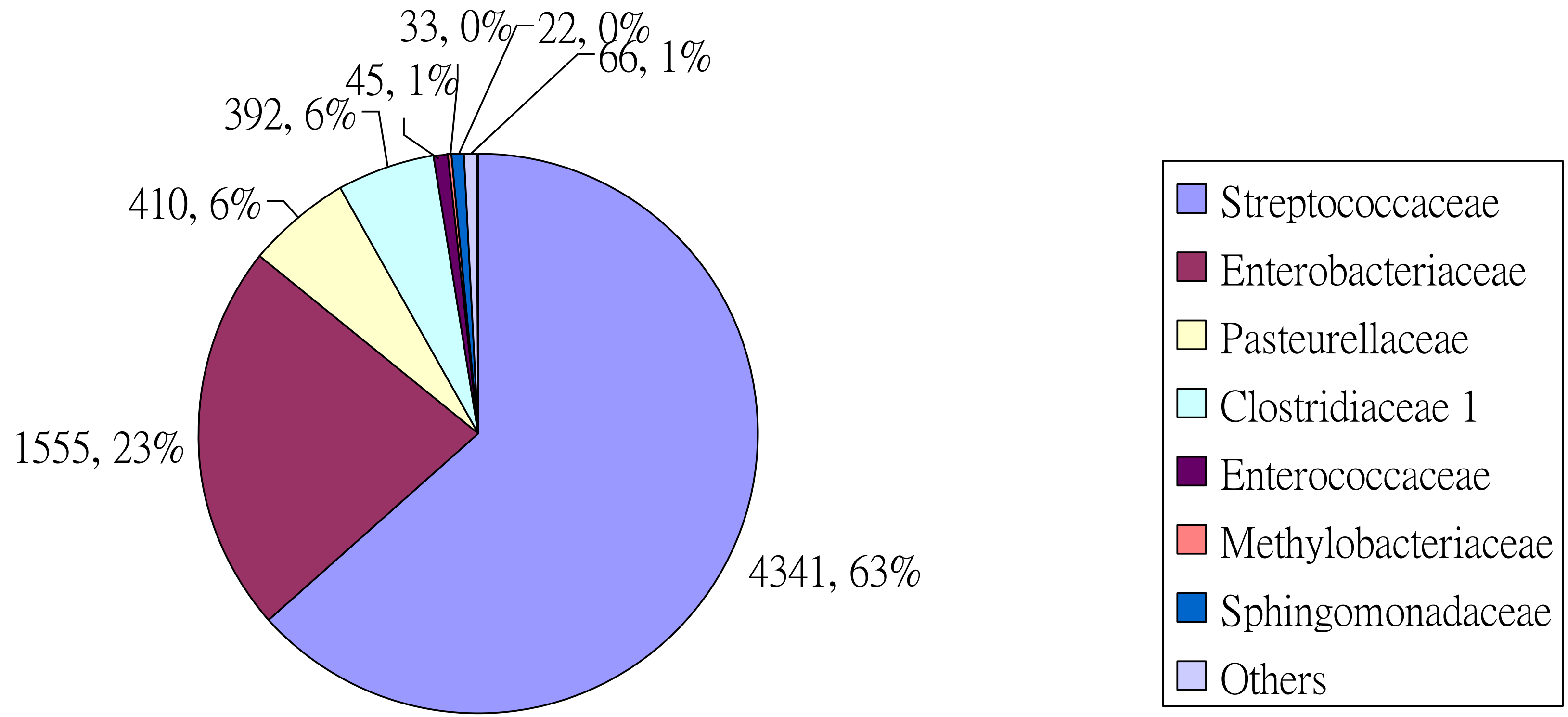

Intestinal microbiota in a 37-day-old neonate with noroviral AGE and necrotizing enterocolitis.

sFig. 1B

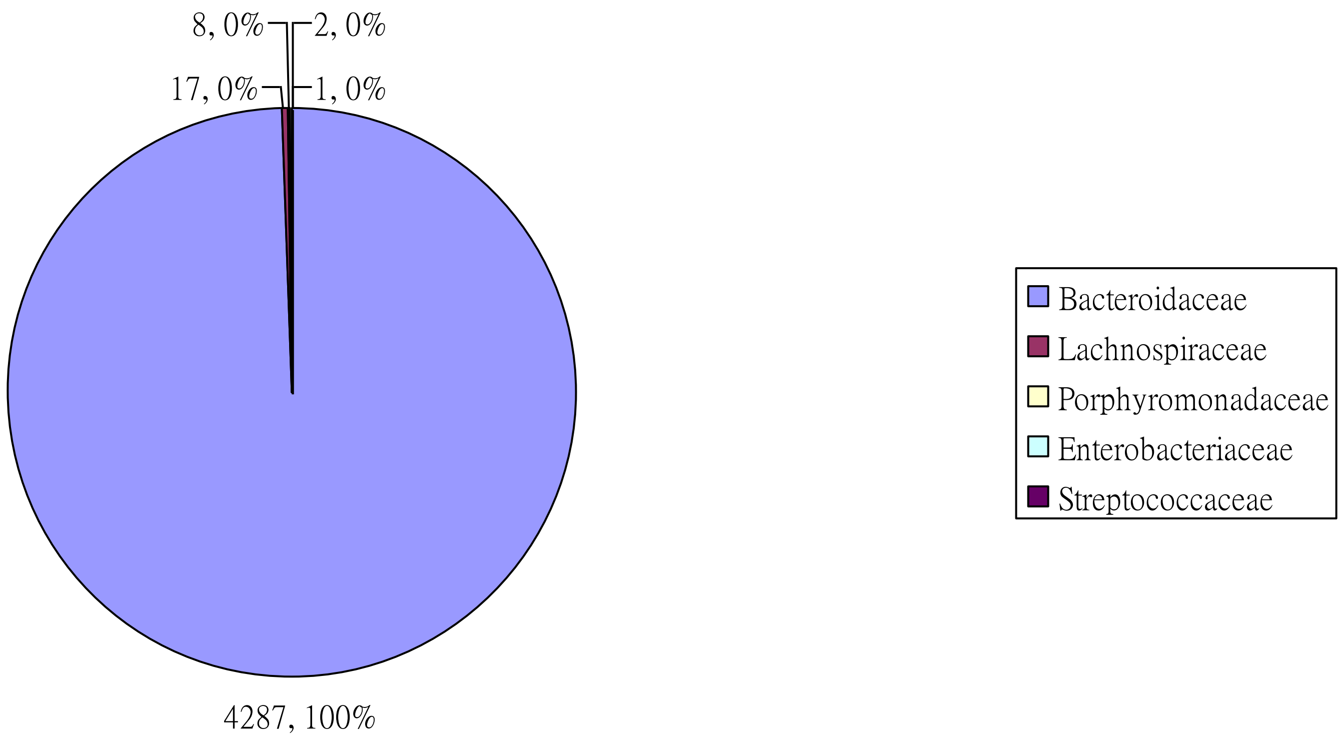

Intestinal microbiota in a 9-month-old male children with rotavirus AGE and convulsion.

sFig. 1C

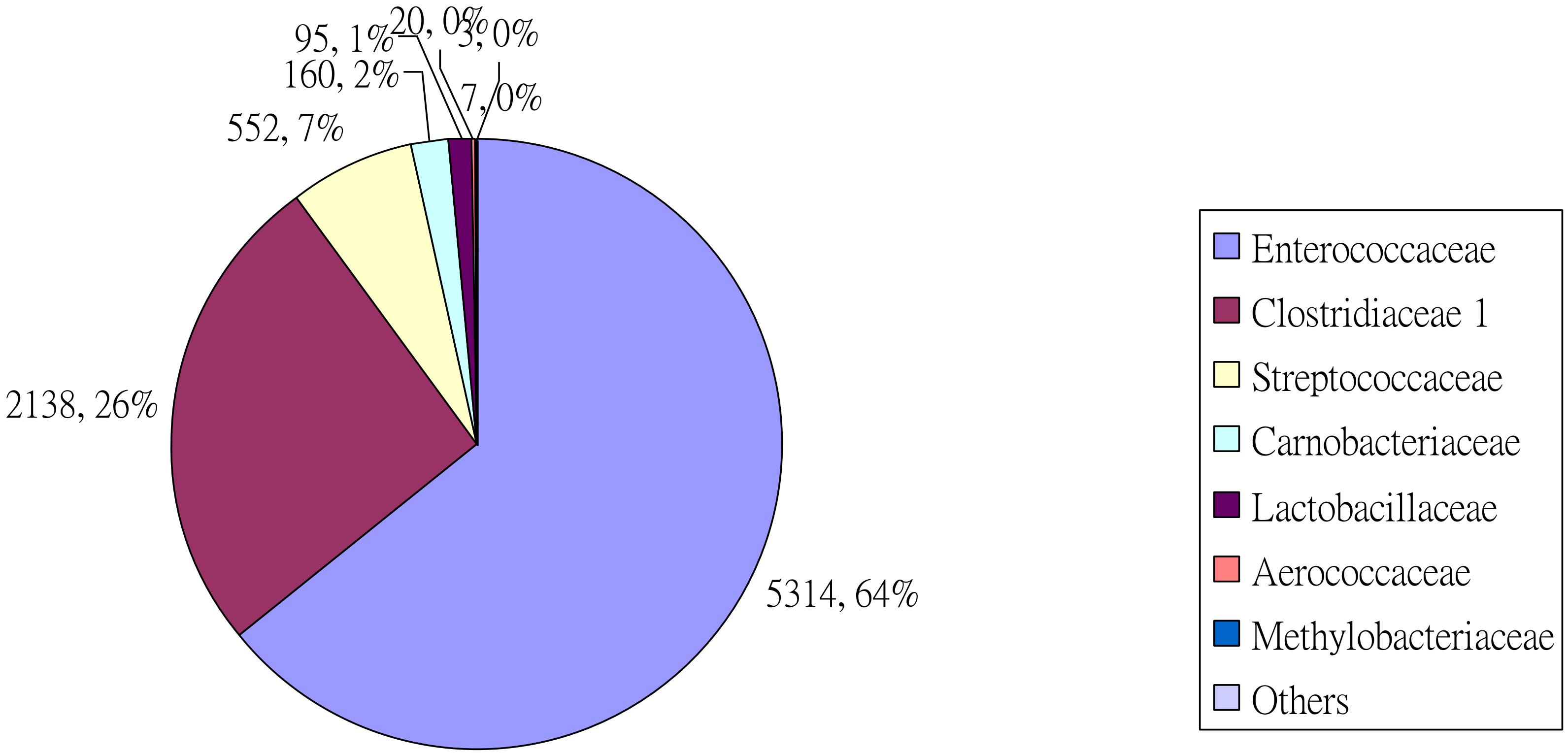

Intestinal microbiota in an 11-month-old male children with noroviral AGE and acute renal failure.
